# CDK16 as a potential prognostic biomarker correlated with an immunosuppressive tumor microenvironment and benefits in enhancing the effectiveness of immunotherapy in human cancers

**DOI:** 10.18632/aging.205465

**Published:** 2024-01-22

**Authors:** Juntao Qi, Gujie Wu, Min He, You Xu, Zheng Yang, Liang Ding, Yan Wang, Zhi Zhang

**Affiliations:** 1 Department of Urology, Shenzhen Hospital of Shanghai University of Traditional Chinese Medicine, Shenzhen 518000, China; 2 Department of Health Management, The First Hospital of Hunan University of Chinese Medicine, Changsha, Hunan 410000, China; 3 Research Center of Clinical Medicine, Affiliated Hospital of Nantong University, Nantong, Jiangsu 226000, China

**Keywords:** CDK16, prognosis, immunosuppressive tumor microenvironment, immunotherapy, human cancers

## Abstract

Background: Cyclin-Dependent Kinase 16 (CDK16) plays significant biological roles in various diseases. Nonetheless, its function in different cancer types and its relationship with the Tumor Immune Microenvironment (TIME) are still not well-understood.

Methods: We analyzed the expression profile, genetic alterations, clinical features, and prognostic value of CDK16 in pan-cancer using data from The Cancer Genome Atlas, Genotype-Tissue Expression databases, and *in vitro* experiments. Additionally, the TIMER2 and ImmuCellAI databases were utilized to assess the correlation between CDK16 expression and immune cell infiltration levels. Finally, we examined the correlation between CDK16 and the response to immunotherapy using collected immunotherapy data.

Results: CDK16 is notably overexpressed in pan-cancer and is a risk factor for poor prognosis in various cancers. Our findings reveal that CDK16 regulates not only cell cycle-related functions to promote cell proliferation but also the autoimmunity-related functions of the innate and adaptive immune systems, along with other immune-related signaling pathways. Moreover, CDK16 overexpression contributes to an immunosuppressive tumor microenvironment, extensively suppressing immune-related features such as the expression of immune-related genes and pathways, as well as the count of immune-infiltrating cells. Our analysis indicated that individuals with low CDK16 expression showed higher response rates to immune checkpoint inhibitors and longer overall survival compared to those with high CDK16 expression.

Conclusions: This study establishes CDK16 as a potential biomarker for predicting poor prognosis in a wide range of cancers. Its role in shaping the immunosuppressive tumor microenvironment and influencing the efficacy of immunotherapy highlights the urgent need for developing targeted therapies against CDK16, offering new avenues for cancer treatment and management.

## INTRODUCTION

Malignant neoplasms, a major health concern, are leading causes of mortality worldwide, placing a significant strain on healthcare systems [1]. Even with substantial progress in cancer treatments, the prognosis for various cancers remains a critical issue, reflecting gaps in our current therapeutic approaches [2, 3]. Central to this challenge is the tumor microenvironment (TME), including the tumor immune microenvironment (TIME), which is crucial in cancer progression and metastasis [4]. Understanding and targeting the complexities of TME and TIME is essential for developing effective cancer treatments and improving patient outcomes [5].

Cyclin-dependent kinase 16 (CDK16) is a distinct member of the “cyclin-dependent kinase” (CDK) family, playing a crucial role in various cellular processes. These include cell cycle regulation, vesicle trafficking, spindle orientation, skeletal myogenesis, neurite outgrowth, transportation of secretory cargo, spermatogenesis, glucose transport, as well as in the regulation of cell apoptosis, growth, proliferation, metastasis, and autophagy [6]. Located on chromosome Xp11.3 in humans, CDK16 is associated with X-linked congenital diseases [7]. Commonly expressed in mammalian tissues, it often acts as an oncogene [8]. As a PCTAIRE kinase, its activity is modulated by the binding of Cyclin Y or its homolog Cyclin Y-like 1 to the N and C-terminal regions of CDK16 [7, 9]. Crucially, CDK16 has been identified as playing a pivotal role in the progression of various cancers, including lung, prostate, breast, melanoma, and hepatocellular carcinomas [10–14]. Despite its significance, the influence of CDK16 on the tumor microenvironment, particularly its correlation with immune therapies, has been historically overlooked. This study conducts a comprehensive analysis of CDK16’s role in pan-cancer and its function within the TME, thereby highlighting its considerable potential in cancer therapy.

In our study, we examined CDK16’s role in various cancers, focusing on its expression, genetic variations, and prognostic relevance. Using data from diverse databases for 33 cancer types, we applied methods like GSEA to assess CDK16’s impact on tumor progression and its connection to immune pathways. We explored the relationship between CDK16 expression, immune cell infiltration, and immune gene expression. Our results highlight a strong link between increased CDK16 expression and an immunosuppressive tumor microenvironment, implying CDK16’s role in tumor-induced immunosuppression and potential implications for immune therapy. This underscores CDK16’s importance in cancer progression and its potential influence on immune therapy outcomes.

## MATERIALS AND METHODS

### Data source

RNAseq data and clinical information data for pan-cancer were obtained from The Cancer Genome Atlas (TCGA) and Genotype-Tissue Expression (GTEx), which were downloaded from UCSC Xena (https://xenabrowser.net/datapages/). Immunocell infiltration data for CDK16 were downloaded from TIMER2 (http://timer.cistrome.org/) and ImmuCellAI (http://bioinfo.life.hust.edu.cn/ImmuCellAI#!/) databases.

### GSEA

To assess the link between CDK16 and its associated genes in human cancers, we employed Gene Set Enrichment Analysis (GSEA). For this analysis, we utilized Pearson correlation coefficients and the R package called “clusterProfiler”. Through these comprehensive analyses, we were able to obtain valuable insights into the relationship between CDK16 and the expression patterns of its associated genes in human cancers.

### Immune infiltration analysis

The R package “ESTIMATE” was used to assess the TME in pan-cancer. Immune cell infiltration data were obtained from the TIMER2 database and published study (Zeng et al., 2019). CDK16 expression and immune cell infiltration levels were correlated to explore the relationship between CDK16 and immune cell infiltration.

### Cell culture

The lung cancer cell line H1299 was obtained from ATCC (Manassas, VA, USA). The cells were cultured in RPMI-1640 medium (Gibco, China) supplemented with 10% fetal bovine serum (Gibco, China). The cultures were maintained at 37°C in a humidified atmosphere containing 5% CO2.

### CCK-8 assay

To assess cell viability, the CCK-8 assay was employed. After transfecting the cells with siRNA for 48 hours, they were transferred to 96-well plates at a density of 3000 cells per well and cultured as described above. CCK-8 reagent (Beyotime, Shanghai, China) was added to each well and cultured for two hours. The optical density at 450 nm was measured at 0, 24, 48, and 72 hours using an iD3 microplate reader.

### RNA extraction and qRT-PCR

According to the manufacturer’s protocol, total RNA was isolated using TRIzol reagent (Pufei, Shanghai, China). The primers sequences used for qRT-PCR were obtained from Applied Biosystems (Ribo, Guangzhou, China) as below (5′–3′): CDK16 forward: 5′-TTGGGCCGTTGGCTGTTC-3′, reverse: 5′-GTGCTCACGGCGGCTC-3′; GAPDH forward: 5′-CAGTGCCAGCCTCGTCTAT-3′, reverse: 5′-AGGGGCCATCCACAGTCTTC-3′. Relative expression levels were calculated according to the 2−ΔΔCt method. Statistical analyses were performed with GraphPad Prism.

### Statistical analysis

We used *t*-tests to assess the disparity in CDK16 gene expression levels between cancer tissues and normal tissues. Survival impacts were analyzed using the Kaplan-Meier method and log-rank test, reporting hazard ratios, confidence intervals, and *p*-values for clarity. Correlation analyses were conducted with Spearman’s or Pearson’s tests based on data type. All analyses were performed using R software (version 4.0.2), with a significance threshold set at a *p*-value of less than 0.05, ensuring the reliability of our findings.

### Data availability statement

The original data supporting the conclusions of this paper will be provided by the authors without reservation.

## RESULTS

### Expression of CDK16 in pan-cancer

**Figure 1:**
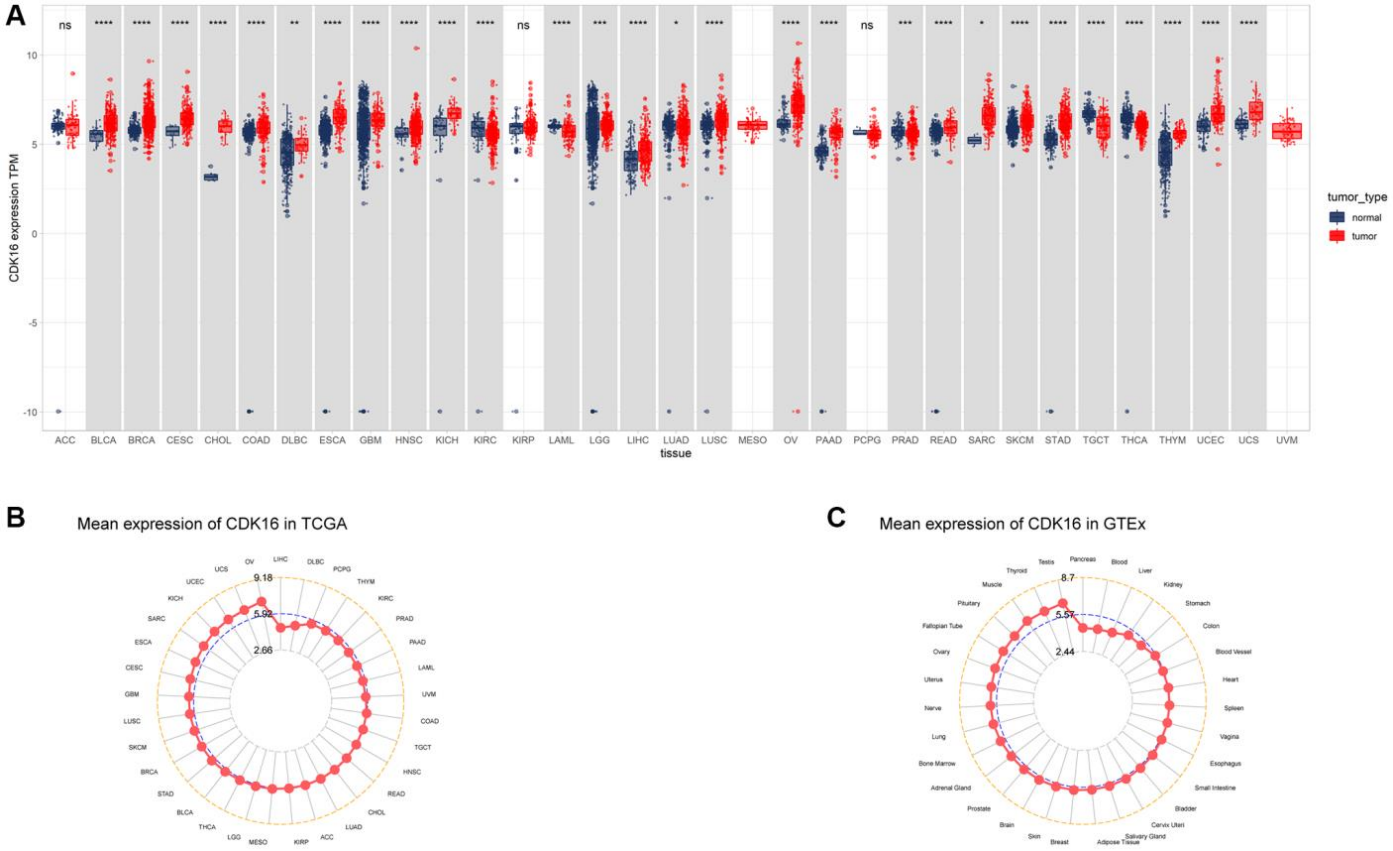
**Expression of CDK16 in pan-cancer.** (**A**) Pan-cancer expression of CDK16. (**B**) CDK16 expression in tumor tissues from TCGA cohort. (**C**) CDK16 expression in normal tissues from GTEx cohort. ^*^P < 0.05, ^**^P < 0.01, ^***^P < 0.001, ^****^P < 0.0001.

**Figure 2:**
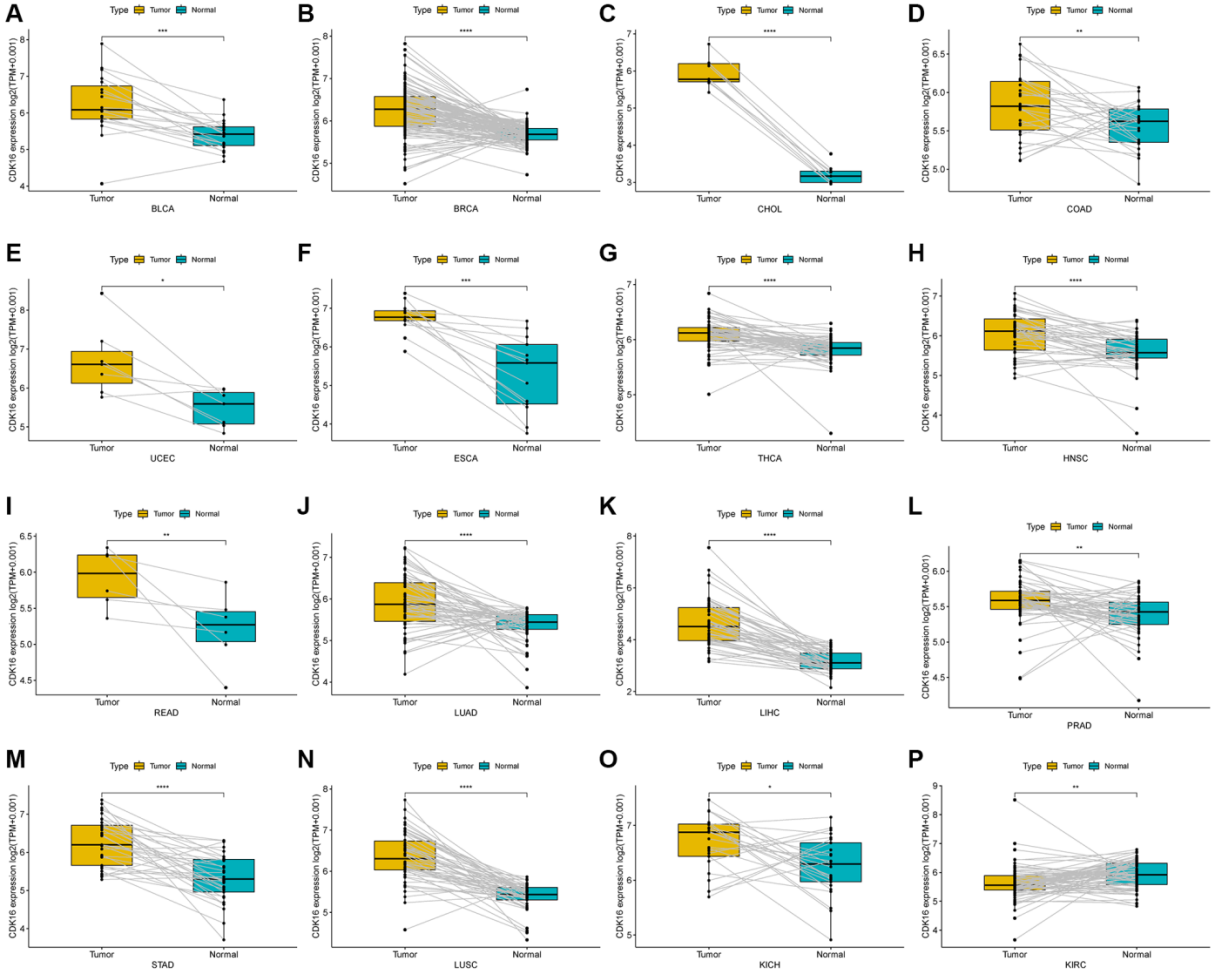
**Expression of CDK16 in paired tumor and adjacent normal tissues.** (**A**–**P**) CDK16 expression in paired tumor and adjacent normal tissues from TCGA in indicated tumor types. ^*^P < 0.05, ^**^P < 0.01, ^***^P < 0.001, ^****^P < 0.0001.

In our study, we assessed CDK16 expression in a pan-cancer context using data sourced from The Cancer Genome Atlas (TCGA) and the Genotype-Tissue Expression (GTEx) project. Our findings revealed that CDK16 was notably overexpressed in 23 out of 33 cancer types examined. These included Bladder Urothelial Carcinoma (BLCA), Breast Invasive Carcinoma (BRCA), Cervical Squamous Cell Carcinoma and Endocervical Adenocarcinoma (CESC), Cholangiocarcinoma (CHOL), Colon Adenocarcinoma (COAD), Lymphoid Neoplasm Diffuse Large B-cell Lymphoma (DLBC), Esophageal Carcinoma (ESCA), Glioblastoma Multiforme (GBM), Head and Neck Squamous Cell Carcinoma (HNSC), Kidney Chromophobe (KICH), Lower Grade Glioma (LGG), Liver Hepatocellular Carcinoma (LIHC), Lung Adenocarcinoma (LUAD), Lung Squamous Cell Carcinoma (LUSC), Ovarian Serous Cystadenocarcinoma (OV), Pancreatic Adenocarcinoma (PAAD), Rectum Adenocarcinoma (READ), Sarcoma (SARC), Skin Cutaneous Melanoma (SKCM), Stomach Adenocarcinoma (STAD), Thymoma (THYM), Uterine Corpus Endometrial Carcinoma (UCEC), and Uterine Carcinosarcoma (UCS). Conversely, CDK16 exhibited lower expression levels in five cancer types, specifically Kidney Renal Clear Cell Carcinoma (KIRC), Acute Myeloid Leukemia (LAML), Prostate Adenocarcinoma (PRAD), Testicular Germ Cell Tumor (TGCT), and Thyroid Carcinoma (THCA), as delineated in Figure 1A and Supplementary Table 1. Notably, the highest expression of CDK16 was observed in ovarian tumor tissues (OV), with the lowest in liver hepatocellular carcinoma (LIHC), as shown in Figure 1B. In normal tissues, the highest expression of CDK16 was identified in the testis (Figure 1C). Additionally, a comparative analysis between tumor tissues and adjacent normal tissues revealed that CDK16 was overexpressed in 15 cancer types, specifically BLCA, BRCA, CHOL, COAD, UCEC, ESCA, THCA, HNSC, READ, LUAD, LIHC, PRAD, STAD, LUSC, and KICH, while it showed lower expression in KIRC, as illustrated in Figure 2A–2P. This differential expression pattern of CDK16 highlights its potential as a biomarker for cancer diagnosis and as a therapeutic target, particularly in cancers with significant overexpression.

### Prognostic role of CDK16

**Figure 3:**
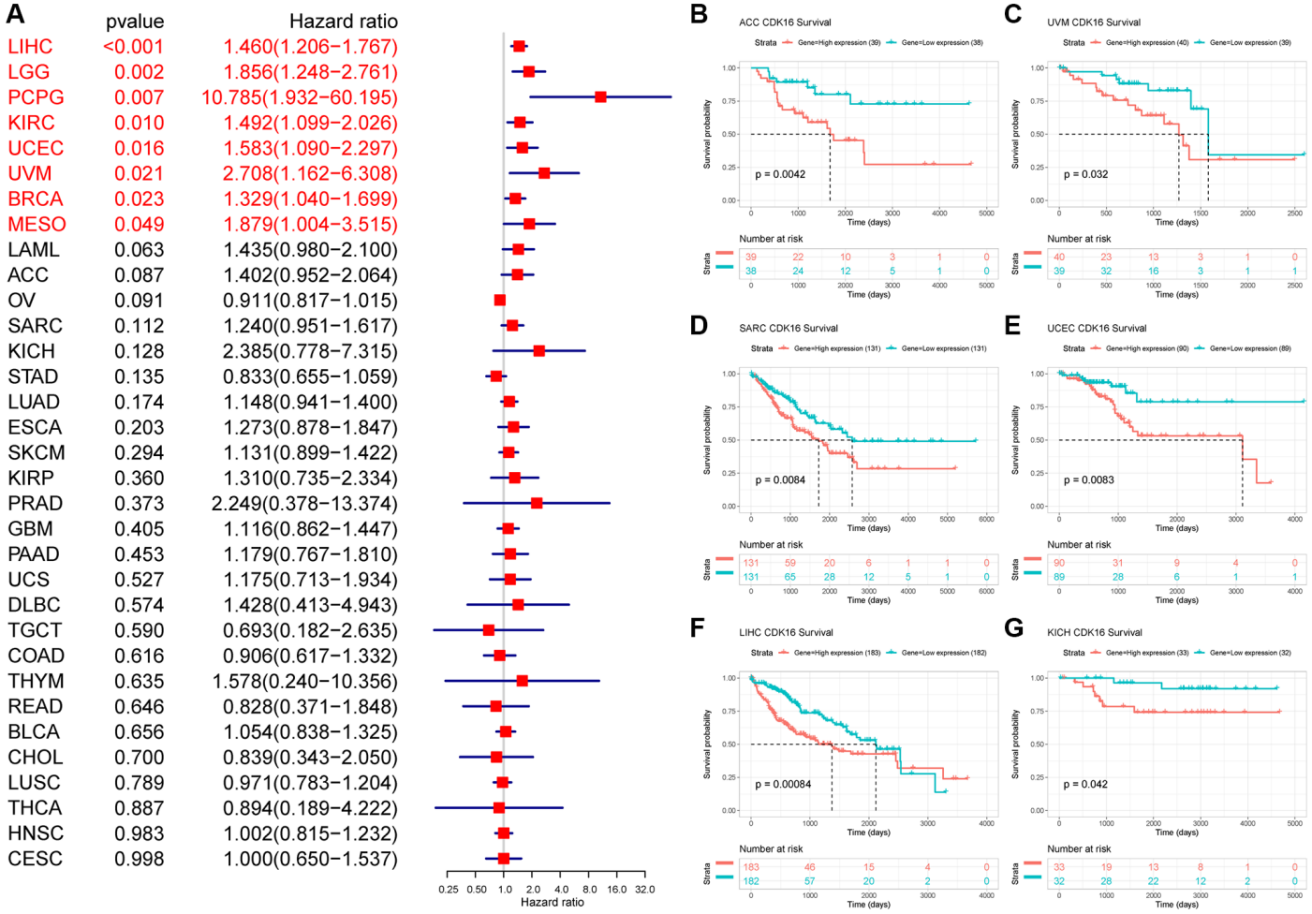
**Relationship between CDK16 level and OS.** (**A**) The univariate Cox regression OS analysis of CDK16 in TCGA pan-cancer. Red color represents significant results (*p* < 0.05). (**B**–**G**) Kaplan-Meier curves showing OS in pan-cancer.

**Figure 4:**
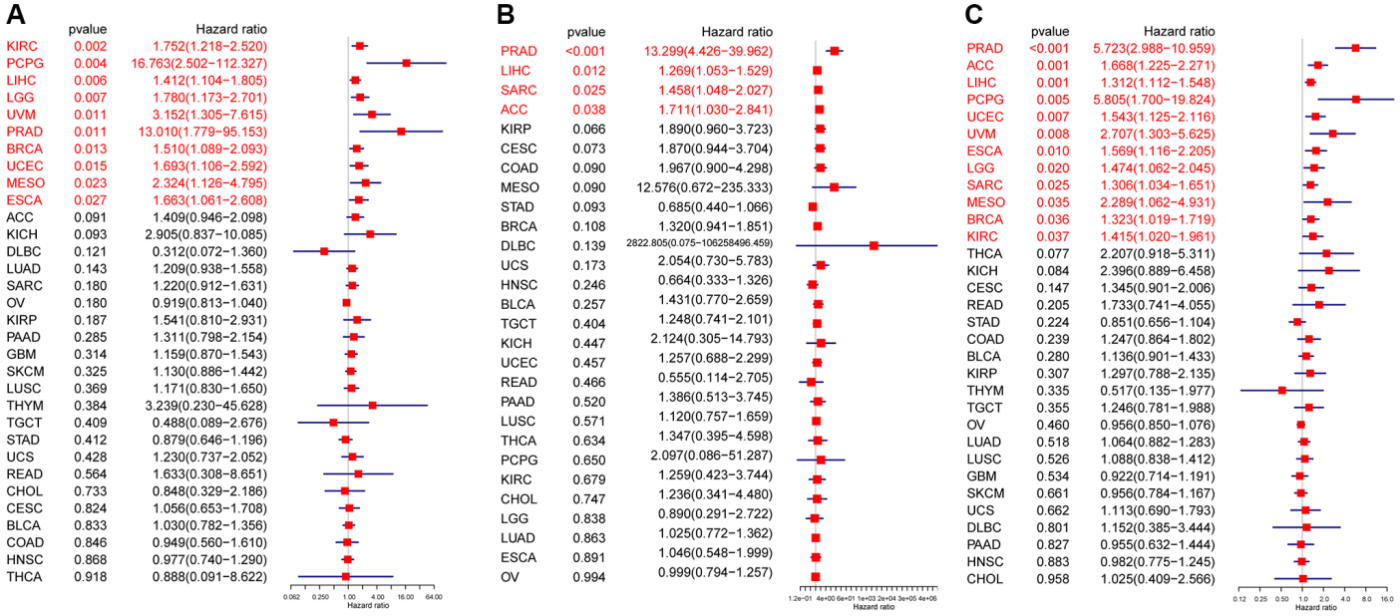
**Prognostic value of CDK16.** Forest plots showing results of univariate Cox regression analysis for (**A**) DSS, (**B**) DFI, and (**C**) PFI. Red color represents significant results (*p* < 0.05).

In the univariate Cox regression analysis (UniCox), CDK16 emerged as a significant risk factor for overall survival (OS) in a range of cancers, namely LIHC, LGG, PCPG, KIRC, UCEC, UVM, BRCA, and MESO (Figure 3A). This finding indicates the potential of CDK16 as a prognostic marker across these diverse cancer types. Kaplan-Meier survival analysis further revealed a correlation between high CDK16 expression and poorer OS in ACC, UVM, SARC, UCEC, LIHC, and KICH (Figure 3B–3G). This pattern highlights the importance of monitoring CDK16 levels in these specific cancers for better prognostic assessment. Concerning disease-specific survival (DSS) outcomes, elevated CDK16 expression was associated with increased risk in KIRC, PCPG, LIHC, LGG, UVM, PRAD, BRCA, UCEC, MESO, and ESCA (Figure 4A). This suggests that CDK16 could play a significant role in the disease course of these cancers. The study also examined the impact of CDK16 on disease-free interval (DFI). High CDK16 expression was predictive of a shorter DFI in PRAD, LIHC, SARC, and ACC (Figure 4B), indicating its potential as an early marker for recurrence or progression in these cancers. Lastly, for progression-free interval (PFI), increased CDK16 expression was linked to worse PFI in PRAD, ACC, LIHC, PCPG, UCEC, UVM, ESCA, LGG, SARC, MESO, BRCA, and KIRC (Figure 4C). This broad association underscores the potential of CDK16 as a marker for monitoring cancer progression across various types. Overall, the expanded analysis underscores the significance of CDK16 in a variety of cancers, suggesting its potential utility as a biomarker for prognosis and therapeutic targeting.

### Gene alteration of CDK16 in pan-cancer

**Figure 5:**
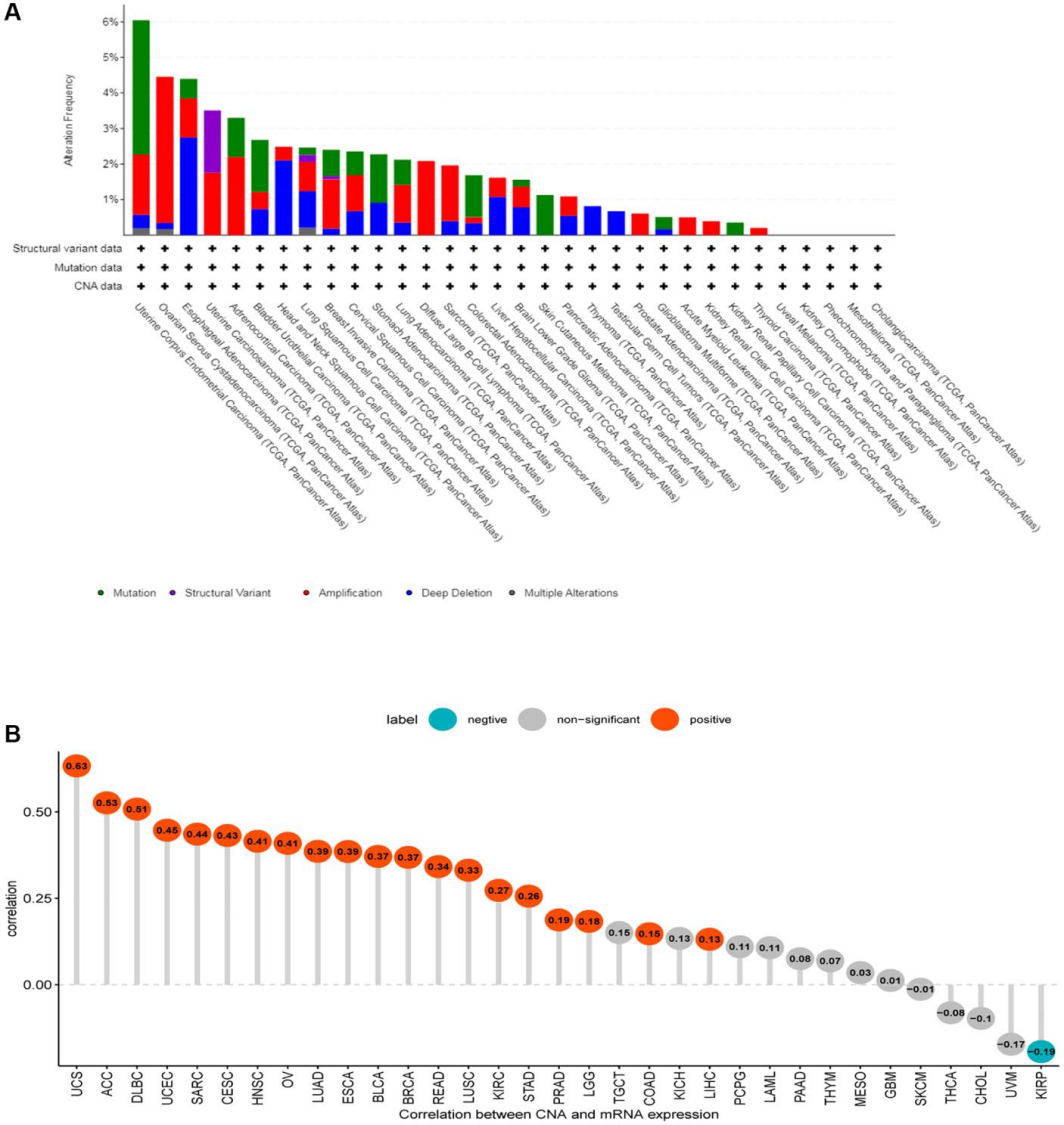
**Gene alteration of CDK16.** (**A**) The mutation and CNA status of CDK16 in TCGA-pan-cancer. (**B**) The correlation between CDK16 expression and CNA.

The established link between genetic alterations and the development of cancer forms the cornerstone of modern oncological research. In our study, we harnessed the advanced data analysis tools provided by cBioPortal to scrutinize the genetic alterations specific to CDK16 across a range of tumor types. Our analysis revealed that the genetic alterations in CDK16 are predominantly in the forms of ‘amplification’ and ‘mutation’ (Figure 5A). This finding not only contributes to the growing body of knowledge about CDK16 but also suggests its potential impact on the pathophysiology of various cancers. Moving beyond this, our investigation also focused on the relationship between CDK16 expression and copy number alterations (CNA). We observed a consistently positive correlation between these two factors across most tumor types we examined (Figure 5B). This correlation is particularly intriguing, as it hints at a more complex role of CDK16 in the genomic landscape of cancer, potentially influencing tumor behavior and response to therapies.

### GSEA analysis of CDK16 in pan-cancer

**Figure 6:**
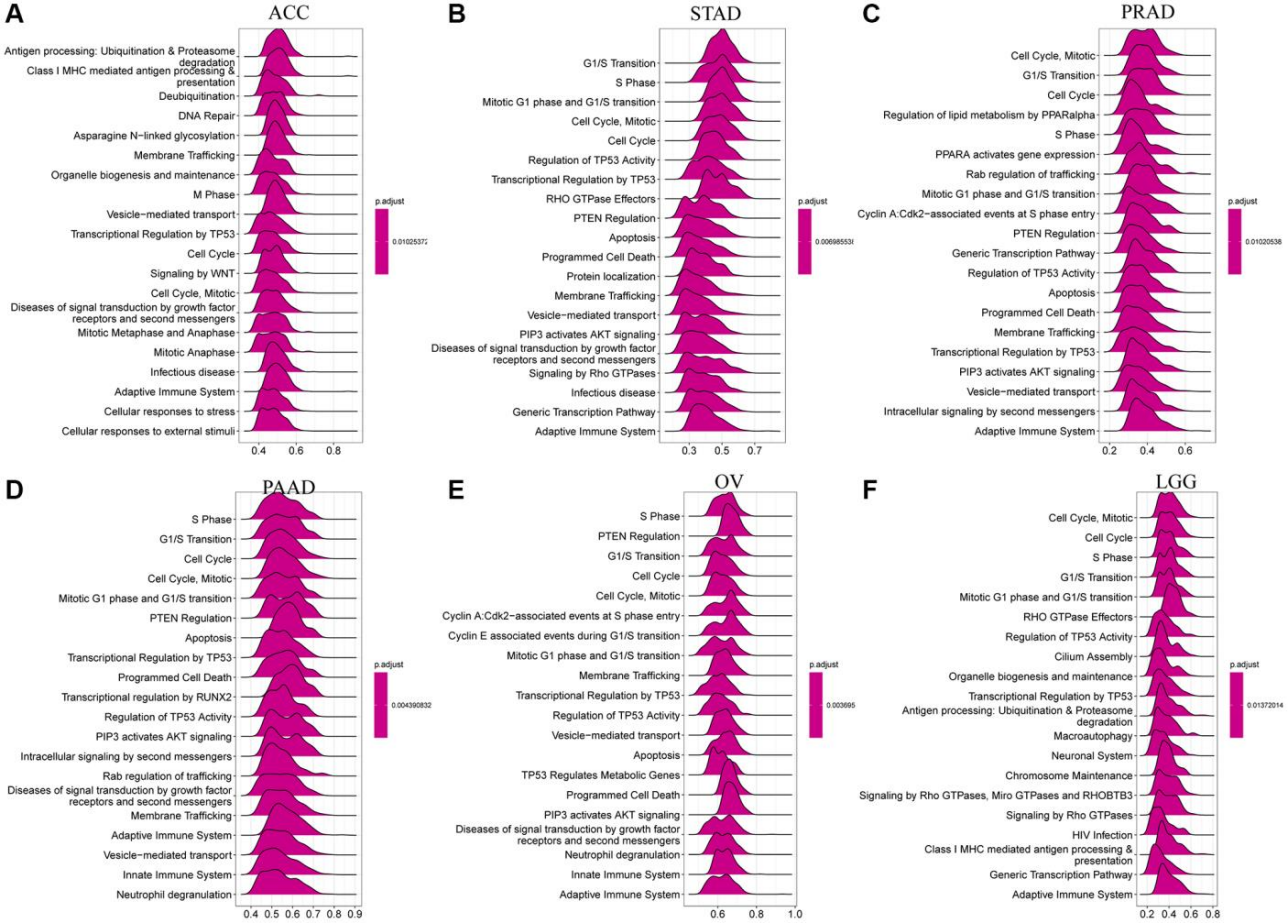
**GSEA of CDK16 in TCGA pan-cancer.** (**A**–**F**) The top 6 significant pathways of GSEA results across the indicated tumor types. Red color represents immune-related pathways.

In an effort to delineate the potential roles of CDK16 in the advancement of tumors, a comprehensive Gene Set Enrichment Analysis (GSEA) was carried out, focusing on the expression levels of CDK16 across a spectrum of 33 distinct cancer types. The results of this analysis are graphically represented in Figure 6A–6F. These findings shed light on the extensive involvement of CDK16 in crucial cell cycle-related pathways. Notably, the pathways include those governing cell cycle regulation and DNA repair mechanisms, which are integral to the proliferation and survival of cancer cells in various types of tumors. The analysis delineates a significant correlation between elevated CDK16 expression and the activation of genes that are pivotal in these pathways. In addition to its prominent role in cell cycle and DNA repair pathways, the study also unveiled that CDK16 is intricately involved in the regulation of immunomodulatory pathways. This encompasses both innate and adaptive immune systems, highlighting a versatile role of CDK16 in modulating the immune response within the tumor microenvironment. The implications of such involvement are twofold: firstly, CDK16’s influence on the immune system may contribute to the immune evasion strategies employed by tumors; secondly, it suggests potential therapeutic targets within these pathways that could be exploited to enhance anti-tumor immunity.

### Relationship between CDK16 and immune cell infiltration

**Figure 7:**
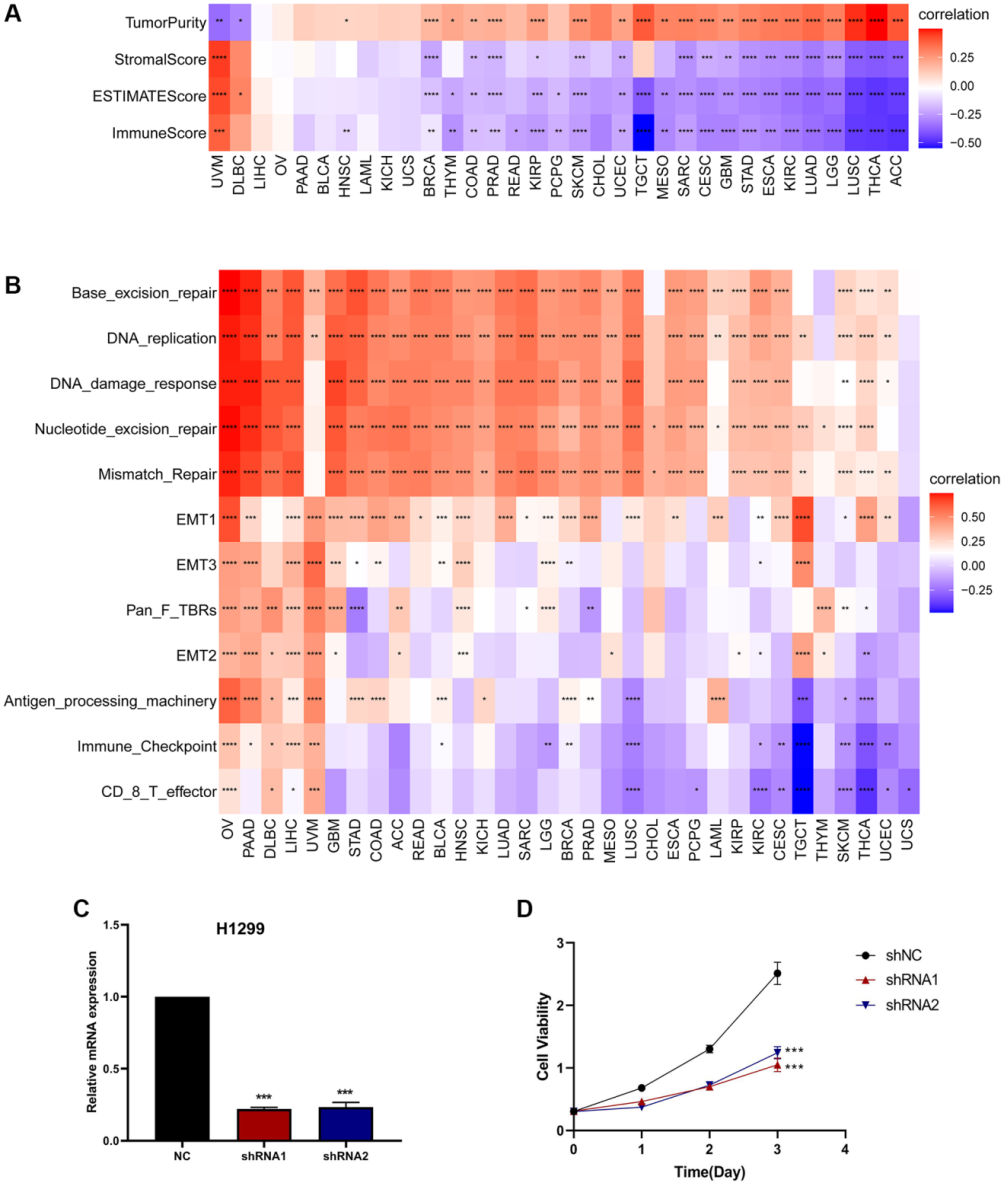
**The relationship between CDK16 and the regulation of the tumour microenvironment.** (**A**) Heatmap represents the correlation between CDK16 expression and TME scores in pan-cancer. (**B**) The relationship between CDK16 and the tumour microenvironment. (**C**) After H1299 cells were transfected with si-CDK16, the level of CDK16 was evaluated by qRT-PCR. (**D**) The cell viability of cells was examined by CCK-8 assay. Red represents positive correlation, blue represents negative correlation, and the darker the color, the stronger the correlation. ^*^P < 0.05, ^**^P < 0.01, ^***^P < 0.001, ^****^P < 0.0001.

We evaluated the association between CDK16 expression and stromal as well as immune scoring, employing the ‘ESTIMATE’ algorithm for this analysis. As depicted in Figure 6, it was observed that CDK16 expression potentially influences immune scores, stromal scores, and ESTIMATE scores in the majority of tumors (Figure 7A). To validate these observations, we further examined the Tumor Microenvironment (TME)-related pathways influenced by CDK16. This included an analysis of immune-related pathways, stroma-related pathways, and DNA repair-related pathways, drawing on data sources from published research [15]. The results corroborated our initial findings, demonstrating a close association between CDK16 expression and various immune-related pathways. These pathways encompass immune checkpoints, antigen processing mechanisms, and CD8 T cell effector functions across a broad range of cancers (Figure 7B).

In conjunction with our GSEA results, these findings collectively provide robust evidence supporting the expansive pro-cancer effects of CDK16. To further substantiate CDK16’s role in promoting cell proliferation, we successfully downregulated its expression in H1299 lung cancer cells using two specific CDK16 shRNAs (Figure 7C). Following this, we conducted Cell Counting Kit-8 (CCK8) assays on these cells. The results from these assays revealed a significant reduction in the viability of H1299 lung cancer cells following CDK16 knockdown (Figure 7D). This outcome lends strong support to our hypothesis that CDK16 functions as an oncogene. Furthermore, when combined with our GSEA results, it becomes evident that CDK16 exerts a broad pro-oncogenic effect across multiple cancer types. Consequently, we proceeded to delve deeper into the mechanistic action of CDK16 in the Tumor Immune Microenvironment (TIME).

**Figure 8:**
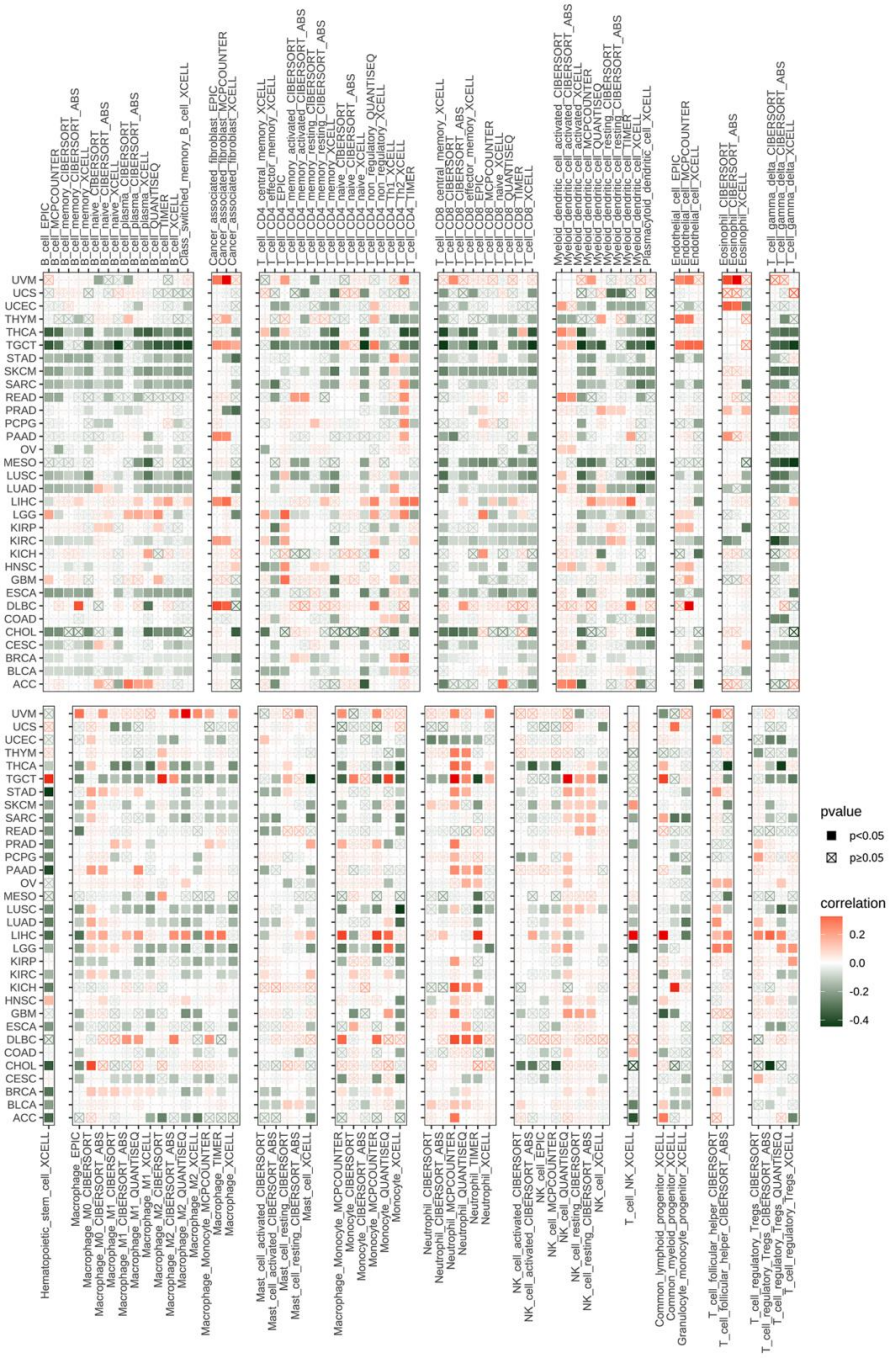
**The relationship between CDK16 and the immune cell infiltration.** Correlation between CDK16 and different immune cells from TIMER2 database.

Dysfunctional immune cell infiltration during tumorigenesis and progression can lead to a loss of immune system control over tumor growth and may even promote tumor development, resulting in immune escape. To explore this further, we utilized immune cell infiltration data from the TIMER2 and ImmuCellAI databases. Our investigation into the relationship between CDK16 expression and immune cell infiltration revealed that high CDK16 expression is associated with significantly poorer immune cell infiltration in pan-cancer (Figure 8 and Supplementary Figure 1).

### Immune-related gene analysis

**Figure 9:**
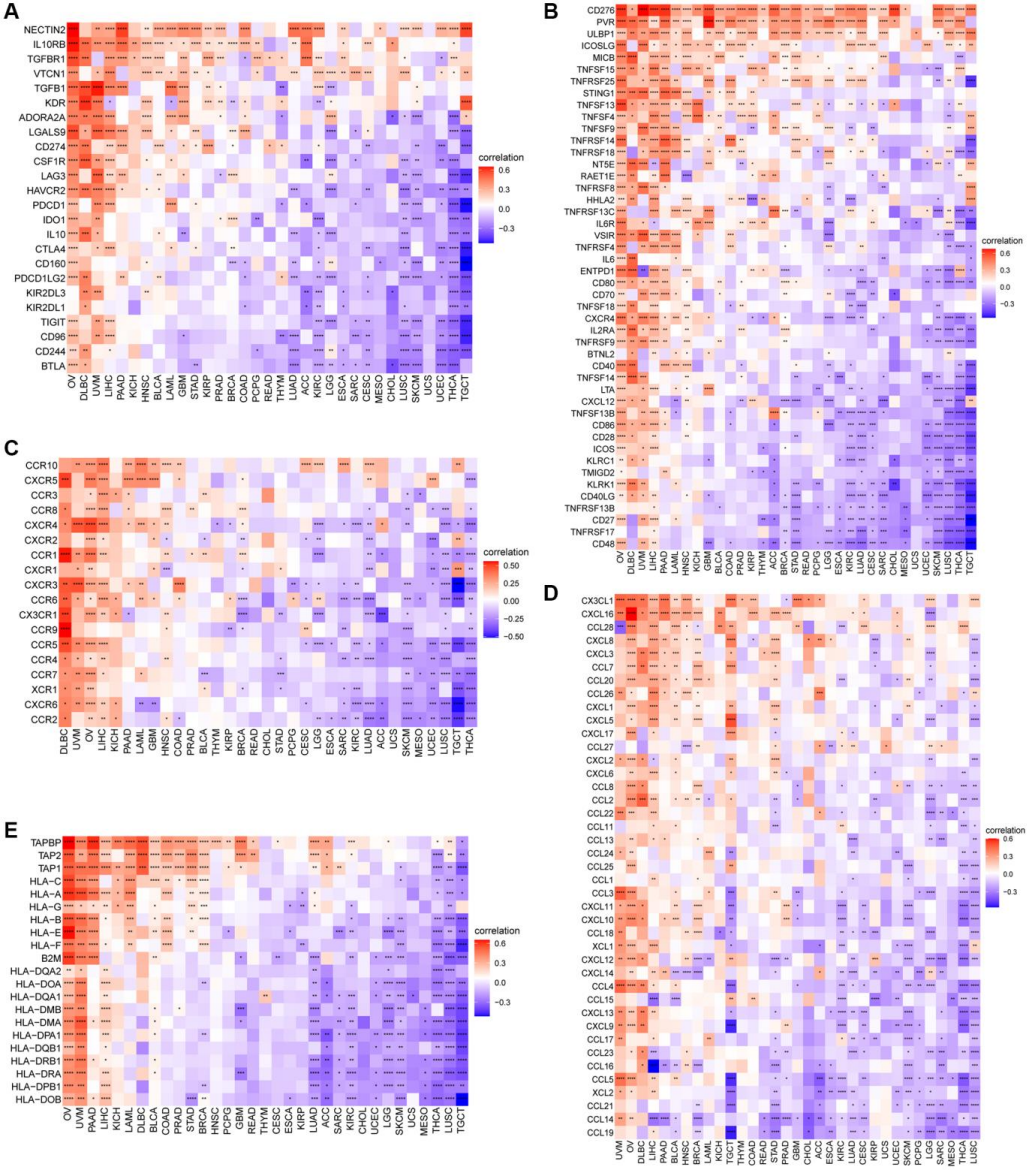
**Relationship between CDK16 expression and that of immune-related genes.** (**A**) Immunosuppressive genes. (**B**) Immune activated genes. (**C**) Chemokines. (**D**) Chemokine receptors. (**E**) MHC genes. Red represents positive correlation, blue represents negative correlation, and the darker the color, the stronger the correlation. ^*^P < 0.05, ^**^P < 0.01, ^***^P < 0.001, ^****^P < 0.0001.

To delve deeper into the intricate relationship between CDK16 and the immunosuppressive microenvironment, we expanded our analysis to include detailed correlation studies between CDK16 and a broad array of immune-related genes. Our investigation revealed a significant association of CDK16 with various immunomodulatory genes. This encompassed not only immunosuppressive genes, as illustrated in Figure 9A, but also extended to genes responsible for immune activation (Figure 9B), an array of chemokines (Figure 9C), a spectrum of chemokine receptors (Figure 9D), and multiple Major Histocompatibility Complex (MHC) genes (Figure 9E). This comprehensive analysis underscores the multifaceted role of CDK16 in modulating the immune landscape. Particularly noteworthy was the finding that elevated levels of CDK16 expression were frequently correlated with a more pronounced immunosuppressive milieu. Such an environment potentially facilitates the evasion of immune surveillance by tumor cells, thus highlighting the pivotal role of CDK16 in the complex interplay between cancer cells and the immune system.

### Immunotherapy

**Figure 10:**
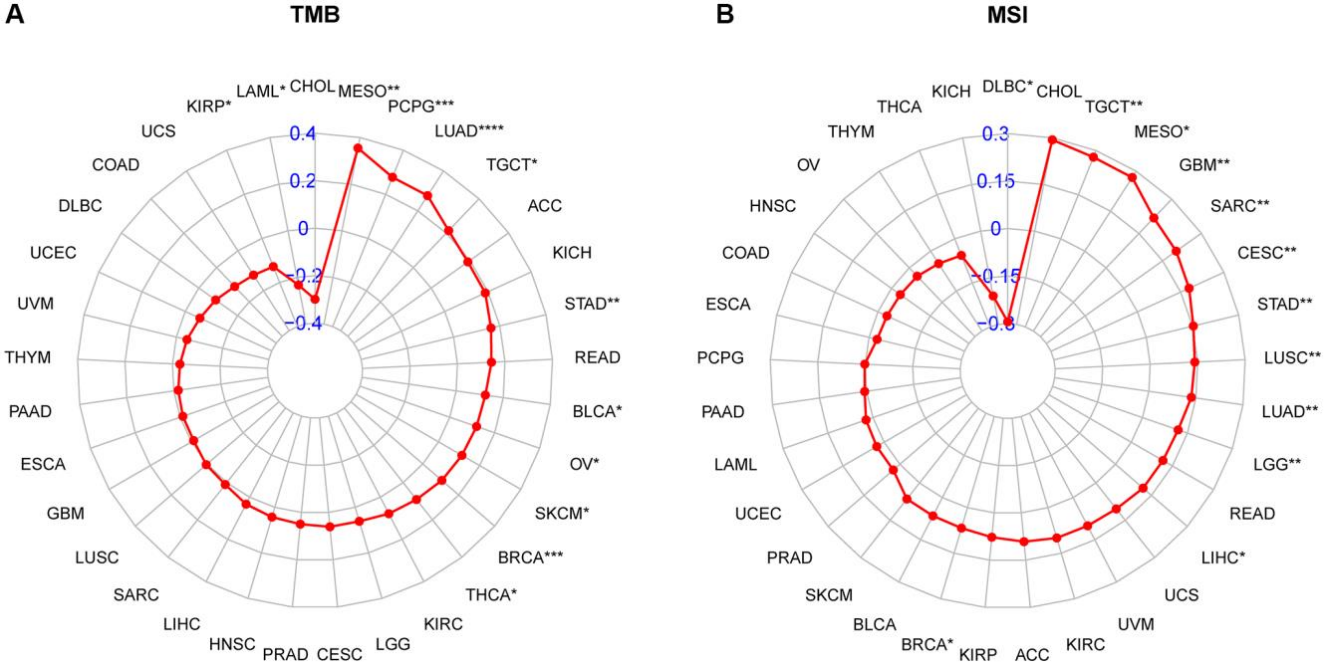
**Relationship of CDK16 expression and tumor mutational burden (TMB), microsatellite instability (MSI).** (**A**) Radar map illustrating the relationship between CDK16 expression and TMB. (**B**) Radar map illustrating the relationship between CDK16 expression and MSI. The red lines represent correlation coefficients, and blue values represent ranges. Spearman correlation test, ^*^p < 0.05, ^**^p < 0.01, ^***^p < 0.001, and ^****^p < 0.0001.

**Figure 11:**
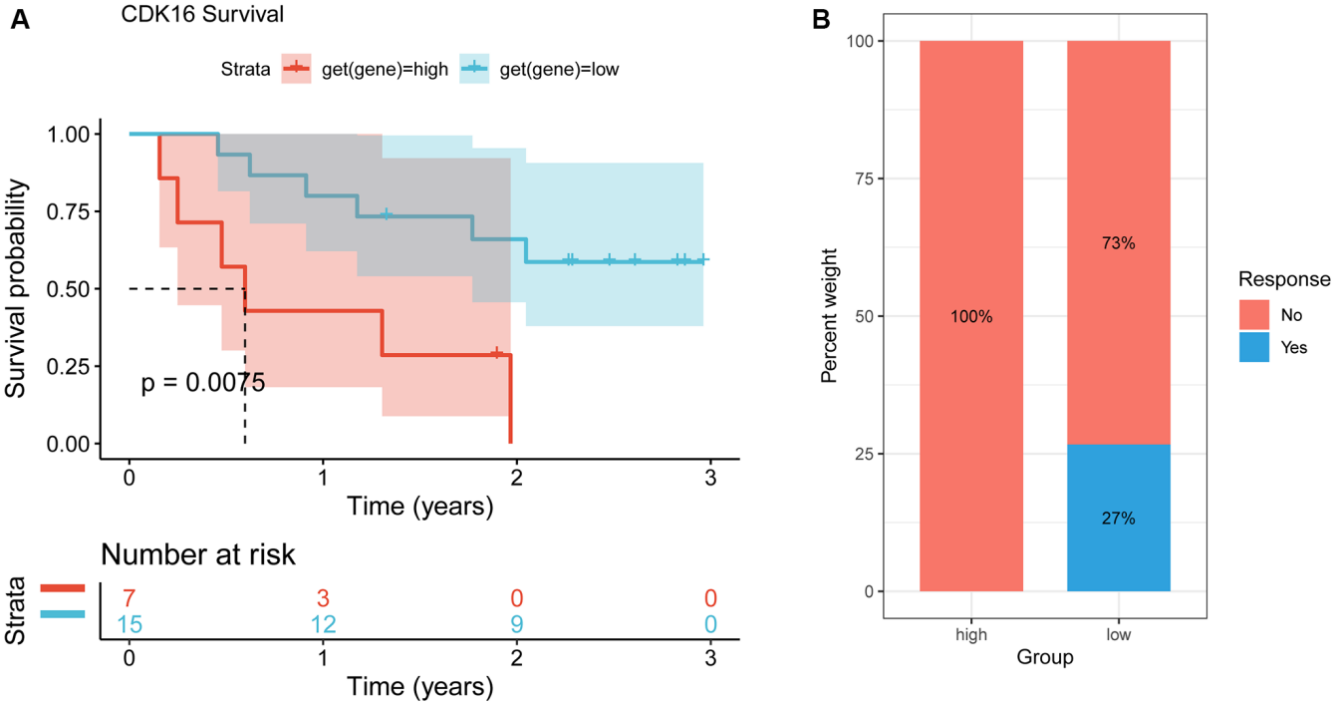
**Correlation between CDK16 and immunotherapy.** (**A**) The Kaplan-Meier overall survival analysis of responsive patients in high- and low- CDK16 expression groups (GSE91061). (**B**) The Kaplan-Meier OS analysis and percentage of responsive patients in high- and low-CDK16 expression groups of GSE91061 cohort.

Immune checkpoint inhibitors (ICIs) have demonstrated potential efficacy in cancer patients exhibiting a high tumor mutation burden (TMB) or microsatellite instability (MSI). Therefore, we investigated the correlation between ICIs and CDK16 expression across multiple cancer types. Figure 6A illustrates a strong positive association between CDK16 expression and TMB in ten cancers, namely malignant MESO, PCPG, LUAD, TGCT, STAD, BLCA, OV, SKCM, BRCA, and THCA (Figure 10A). Similarly, CDK16 expression showed a positive correlation with MSI in eleven cancers, including TCGT, MESO, GBM, SARC, CESC, STAD, LUSC, LUAD, LGG, LIHC, and BRCA (Figure 10B). These findings underscore the potential involvement of CDK16 in immunotherapeutic approaches. Hence, it is reasonable to propose that CDK16 expression may influence the response to ICI treatment and prognosis in patients affected by various cancer types. To test this hypothesis, we collected data from cohorts of patients who underwent immunotherapy and evaluated CDK16 expression across different cancer types. Kaplan-Meier analysis revealed that low CDK16 expression was associated with prolonged overall survival and improved overall response rate in patients treated with nivolumab, a PD-1 inhibitor utilized in cancer immunotherapy (Figure 11A, 11B). These findings emphasize the significant role of CDK16 expression in determining patient response to immunotherapy.

## DISCUSSION

Cancer is a complex and multifaceted disease driven by dysregulation of various cellular pathways, including those involved in cell metabolism, cell death, inflammation, migration, invasion, and immune evasion [16, 17]. The tumor microenvironment (TME) plays a critical role in tumor progression and metastasis, serving as a key mediator of these processes [18, 19]. Additionally, the TME has emerged as a crucial factor in predicting clinical outcomes and determining the response of tumor cells to immunotherapy. Emerging evidence suggests that the immunosuppressive nature of the TME can facilitate tumor growth and dampen the efficacy of immunotherapy in cancer patients [20, 21]. The intricate interplay between tumor cells and the immune system within the TME creates an immunosuppressive state that enables cancer cells to evade immune detection and destruction [22, 23]. Given the significant impact of immunosuppression in cancer, there is an urgent need to identify potential biomarkers that can shed light on the immunological landscape of the TME. These biomarkers could aid in patient stratification, treatment selection, and monitoring of therapeutic response. By uncovering molecular signatures indicative of immunosuppression, researchers and clinicians can develop targeted interventions to reverse immunosuppressive mechanisms and enhance the effectiveness of immunotherapy.

CDK16 plays a crucial role in regulating biological functions rhythmically. However, its role in the initiation and progression of tumors has been largely overlooked [24]. Previous studies have only sparingly assessed the significance of CDK16 in cancer, such as its ability to modulate cancer cell growth and apoptosis through a p27-dependent mechanism [25]. Recognizing the potential importance of CDK16 in cancer, we conducted a comprehensive analysis of CDK16 in a pan-cancer context, investigating its expression, genetic alterations, biological functions, and its relationship with immune regulation and treatment response. Firstly, we observed overexpression of CDK16 in various cancer types, indicating its potential role as a promoter in tumor development. This finding aligns with existing literature supporting the concept of CDK16 as a potential cancer biomarker. Previous research has shown that high CDK16 expression is associated with tumor growth and infiltration, consistent with our findings. Furthermore, our analysis revealed that high CDK16 expression is correlated with poor prognosis, emphasizing the significance of CDK16 as a potential prognostic marker. Elevated CDK16 expression is linked to shorter overall survival and higher recurrence risk, suggesting a critical role for CDK16 in the malignant progression of tumors. This finding is in line with existing literature supporting the importance of CDK16 in assessing the prognosis of cancer patients. The use of CDK16 as a prognostic marker may help guide treatment strategies to improve patient survival. Additionally, we investigated genetic alterations in CDK16. Gene amplification and mutation were identified as the primary types of genetic alterations in CDK16, which may be related to tumor development and treatment response. These genetic alterations may lead to abnormal CDK16 activity, promoting tumor growth. This discovery provides clues for further research into the molecular mechanisms of CDK16 and may serve as a target for novel treatment strategies.

On the other hand, our gene enrichment analysis and immune regulation study revealed the multifaceted role of CDK16. CDK16 is not only involved in crucial pathways such as the cell cycle and DNA repair but also plays a role in regulating immune responses. This suggests that CDK16 may affect immune cell infiltration in the tumor microenvironment, influencing immune responses. This finding is of significant importance for understanding the potential mechanisms of immunotherapy and provides new insights for the development of novel immunotherapeutic strategies. Furthermore, we delved into the relationship between CDK16 and immune therapy, which holds significant importance in the current landscape of cancer immunotherapy. Tumor Mutational Burden (TMB) is widely recognized as a crucial determinant of immune therapy efficacy [26, 27]. Previous research has established that high TMB correlates with improved clinical responses to immune checkpoint inhibitors (ICIs) in patients with melanoma [28], head and neck cancers [29], and urothelial carcinoma [30]. TMB serves as a prognostic and predictive biomarker for human cancer immunotherapy. Microsatellite Instability (MSI) is also a key biomarker for ICI treatment response [31]. Our study has revealed a positive correlation between CDK16 expression levels and TMB as well as MSI, further emphasizing the potential significance of CDK16 in the context of immune therapy. Low CDK16 expression is associated with a more favorable prognosis in immune therapy, suggesting CDK16 as a potential predictive factor for immunotherapy. This finding is poised to provide a solid foundation for the development of personalized immune therapy strategies, aiding in the identification of patients who are likely to benefit from immune therapy.

While this research has provided valuable insights, we acknowledge its limitations. Most of the analyses were conducted using publicly available databases. To validate the research findings, further *in vitro* and *in vivo* experiments, as well as large-scale prospective studies, are required. Future research efforts should aim to delve deeper into and explore the findings of this study.

In conclusion, our research results provide a comprehensive understanding of the role of CDK16 in various cancers and lay the foundation for future research and clinical applications. Further research will help uncover the exact mechanisms of CDK16 and develop new treatment strategies to improve cancer patient outcomes. Additionally, CDK16 as a potential target for immunotherapy warrants further investigation to expand the application of immunotherapy in cancer treatment.

## Supplementary Materials

Supplementary Figure 1

Supplementary Table 1
