# Supplementary material for: CDK16 as a potential prognostic biomarker correlated with an immunosuppressive tumor microenvironment and benefits in enhancing the effectiveness of immunotherapy in human cancers
Source: Aging (Albany NY). 2024 Jan 22;16(2):1879–96. doi: 10.18632/aging.205465 (PMC10866429; doi:10.18632/aging.205465)
Supplement: Supplementary Figure 1 [file aging-16-205465-s001.pdf]

SUPPLEMENTARY FIGURE

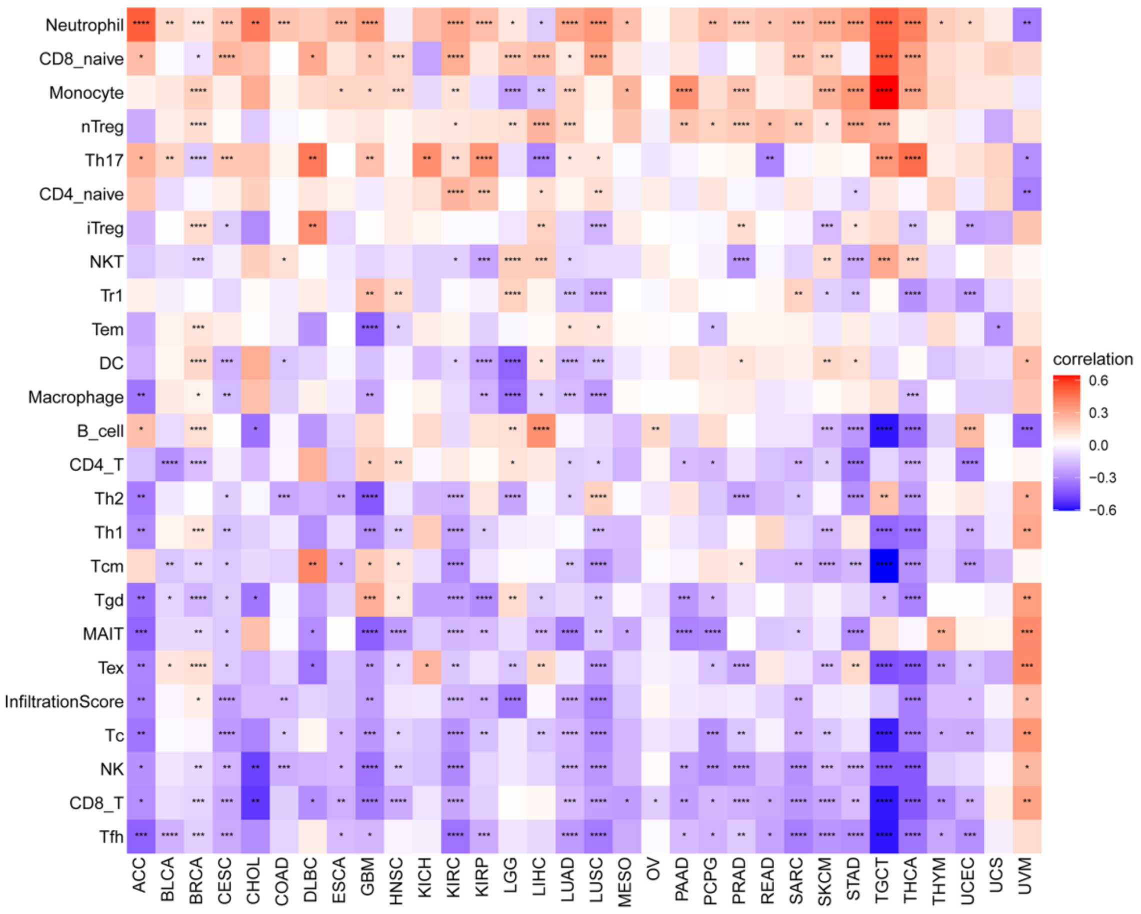

Supplementary Figure 1. Correlation between CDK16 and different immune cells from ImmuCellAI database.
